# Decreasing expression of HIF-1α, VEGF-A, and Ki67 with efficacy of neoadjuvant therapy in locally advanced cervical cancer

**DOI:** 10.1097/MD.0000000000033820

**Published:** 2023-05-17

**Authors:** Ke Zhao, Min Hu, Runfeng Yang, Jing Liu, Pingfan Zeng, Tingkuan Zhao

**Affiliations:** a Department of Gynecologic Oncology, Hubei Cancer Hospital, Tongji Medical College, Huazhong University of Science and Technology, Wuhan, Hubei, China; b Department of Pathology, Hubei Cancer Hospital, Tongji Medical College, Huazhong University of Science and Technology, Wuhan, Hubei, China; c Department of Pathology, Jingzhou Central Hospital, Tongji Medical College, Huazhong University of Science and Technology, Jingzhou, Hubei, China.

**Keywords:** HIF-1α, Ki67, locally advanced cervical cancer (LACC), NACT, VEGF-A

## Abstract

Neoadjuvant chemotherapy (NACT) before radical hysterectomy has been widely used for locally advanced cervical cancer (LACC); However, its efficacy is yet to be determined.

**Methods:**

Effective and predictive biomarkers, which may aid in predicting the chemotherapy responses, were explored in this study. Initially, the expression of HIF-1α, VEGF-A, and Ki67 was detected in 42 paired (pre-NACT and post-NACT) LACC tissues, as well as 40 nonneoplastic cervical epithelial tissues by immunohistochemistry. Then, the correlation of the expression of HIF-1α, VEGF-A, Ki67 with the efficacy of NACT, as well as factors that affect the efficacy of NACT was analyzed.

**Results:**

A clinical response occurred in 66.7% (28/42) of the patients, including 57.1% (16/28) with a complete response and 42.9% (12/28) with a partial response; While 33.33% (14/42) were non-responders, including 42.9% (6/14) with stable disease and 57.1% (8/14) with progressive disease. HIF-1α, VEGF-A, and Ki67 were overexpressed in LACC tissues compared to nonneoplastic tissues (*P <* .01, respectively); While the expression of HIF-1α, VEGF-A, and Ki67 was significantly decreased after NACT (*P* < .01, respectively). What’s more, in the response group, HIF-1α, VEGF-A, and Ki67 expression were significantly decreased after chemotherapy in the post-chemotherapy cervical cancer tissues compared with the pre-chemotherapy cervical cancer tissues (all *P* < .05). Additionally, patients with lower histological grade and lower expression of HIF-1α, VEGF-A, and Ki67 were more responsive to NACT (*P* < .05, respectively); Moreover, the histological grade [P = .025, HR (95% CI): 0.133 (0.023–0.777)], HIF-1α [P = .019, HR (95% CI): 0.599 (0.390–0.918)], and Ki67 [*P* = .036, HR (95% CI): 0.946 (0898–0.996)] were independent risk factors affecting the efficacy of NACT in LACC.

**Conclusion:**

Expression of HIF-1α, VEGF-A, and Ki67 were significantly decreased after NACT, and decreasing expression of HIF-1α, VEGF-A, and Ki67 were related to good response to NACT, suggesting HIF-1α, VEGF-A, and Ki67 may be implicated in evaluating the efficacy of NACT in LACC.

## 1. Introduction

Cervical cancer remains the 4th most common malignancy diagnosed in women globally, despite the several advances in its screening, prevention, diagnosis, and treatment in recent years.^[1]^ More than half a million women are diagnosed with cervical cancer annually, resulting in over 300,000 deaths, with high incidence and mortality in developing countries.^[2]^ Since the early 1980s, neoadjuvant chemotherapy (NACT) was suggested for control of the disease before surgery, or as adjuvant treatment in combination with radiotherapy after surgery.^[3]^ Concurrent chemoradiation was considered instead of radiotherapy alone in women with cervical cancer in 1999^[4]^; However, a subsequent meta-analysis suggested that NACT followed by surgery is superior to radiotherapy alone in terms of overall survival.^[5]^

Platinum-based NACT before radical hysterectomy has been widely used for locally advanced cervical cancer (LACC), which is defined as the International Federation of Gynecology and Obstetrics (FIGO) stage IB2-IIIB for decades. In a large majority of LACC patients treated with NACT, the treatment is effective, such that it can shrink the tumor size and suppress micrometastasis, thus improving prognosis and reducing the risk of recurrence.^[6,7]^ However, in some cases, NACT failed to achieve tumor regression, but the prognosis of these cases would become worse due to delayed curative treatment.^[8]^ Moreover, cancer patients receiving chemotherapy usually experience a wide range of distressing side effects, including anemia, neutropenia, nausea, and neurotoxicity.^[9]^ Therefore, the effect of NACT and predictive biomarkers which may aid in predicting the chemotherapy responses would greatly optimize the treatment options of LACC patients.

Hypoxia plays an important role in regulating tumor progression, metastasis, and resistance to radiochemotherapy in some solid tumors.^[10]^ Hypoxia-inducible factor-1α (HIF-1α), an oxygen-regulating protein, is the major transcriptional factor in regulating oxygen homeostasis.^[11]^ HIF-1α activated by hypoxia or aberrant activation of some oncogenes participates in the malignant transformation of a variety of solid tumors and provides a good prerequisite for tumor growth and metastasis.^[12,13]^ Huang et al^[14]^ demonstrated that overexpression of HIF-1α is more likely to cause metastasis and poor outcome, and could be a predictor of poor prognosis in cervical cancer. Yan et al^[15]^ indicated that the expression level of HIF-1α may be able to predict the efficiency of NACT and may be an independent prognostic factor for stage IIB-IIIB cervical cancer.

As a predominant regulator of intratumoral hypoxia, HIF-1α also controls the angiogenesis of tumors except for carcinogenesis and therapy resistance.^[16,17]^ Angiogenesis, the formation of new blood vessels from the preexisting vessels via sprouting, has been characterized as an essential process for the proliferation and metastasis of tumor cells. Vascular endothelial growth factor (VEGF) is among the most important proangiogenic factors, which plays a critical role in tumor progression and mediates chemoresistance in cervical cancer.^[18]^ Moreover, it has been demonstrated that VEGF and HIF-1α expressions have great clinical significance for the prediction of preoperative radio chemotherapy response and prognosis in patients with LACC.^[19]^ Ki67 is closely related to the carcinogenesis of cervical cancer and should be a biomarker of cervical cancer.^[20]^ However, the efficacy of NACT in LACC, and whether HIF-1α, vascular endothelial growth factor (VEGF-A), and Ki67 could be biomarkers for predicting the chemotherapy responses in LACC remains to be elucidated.

Considering the above background, we hypothesized that NACT could result in decreasing expression of HIF-1α, VEGF-A, and Ki67, and decreased expression of HIF-1α, VEGF-A, and Ki67 was related to good response to NACT in LACC. Our present study supported the view that expression of HIF-1α, VEGF-A, and Ki67 may be implicated in evaluating the efficacy of NACT in LACC.

## 2. Materials and methods

### 2.1. Patients

This was a retrospective study of a series of 42 LACC patients who underwent a hysterectomy after NACT between May 2015 and December 2019 in Jingzhou Central Hospital, Tongji Medical College, Huazhong University of Science and Technology. Inclusion criteria: all patients were cervical squamous cell carcinoma, all patients were first diagnosed with LACC and have both biopsy and surgical specimens; the patients did not receive chemotherapy, radiotherapy, immunotherapy, or hormonal therapy before biopsy; the patients were treated with NACT and then underwent a hysterectomy. Exclusion criteria: recurrent cervical cancer, previous radiotherapy, or combined with other diseases, such as malignant tumors, systemic immune diseases, infectious diseases, organ failure diseases, and cancer complications. A total of 40 nonneoplastic cervical epithelial tissues were selected and included as the control group. The clinicopathological features of the patient were obtained from the Department of Gynecology, Jingzhou Central Hospital, Tongji Medical College, Huazhong University of Science and Technology. The stage of LACC was determined according to FIGO guidelines. The age of the patients was 49 (34–65) years. The maximum diameter of the tumor was ≤ 4cm in 25 cases and > 4 cm in 17 cases. Histological classification showed high differentiation in 11 cases, moderate differentiation in 21 cases, and low differentiation in 10 cases. FIGO stage IB2 of 10 cases, IIA of 20 cases, and IIB of 12 cases. There were 32 cases without lymph node metastasis and 10 cases with lymph node metastasis. The study was approved by the Medical Ethics Committee of Jingzhou Central Hospital, Tongji Medical College of Huazhong University of Science and Technology (2020123001), and informed consent for the use of tissues for *ex vivo* experiments was obtained from each patient.

### 2.2. NACT and therapeutic effect evaluation

The chemotherapy regimen of the patients who received NACT was: paclitaxel 135 to 175 mg/m2 followed by cisplatin 70 to 75 mg/m2 on day 1 intravenously, every 21 days for 2 to 3 consecutive courses. Most patients received 3 courses of NACT, and only 2 patients received 2 courses of NACT for physical grounds. The chemotherapy response after the second or third cycle of chemotherapy was evaluated by measuring the tumor’s largest diameter with imaging. The chemotherapy response was assessed 2 weeks after completion of the NACT regimen, according to previous criteria.^[21]^ Complete response (CR) was defined as complete remission of the tumor; Partial response (PR) was defined as at least a 50% decrease in the tumor volume; The patients with CR or PR were defined as chemotherapy responders. Stable disease (SD) implies a steady state or response of <50% and progressive disease (PD) was defined as an unequivocal increase of at least 25% in the tumor volume. The patients with SD or PD were classified into chemotherapy non-responders. Two weeks after completion of the NACT regimen, all 42 patients received radical hysterectomy and bilateral pelvic lymphadenectomy.

### 2.3. Immunohistochemistry

The expression of HIF-1α, VEGF-A, and Ki67 was detected by conventional immunohistochemical (IHC) staining protocol. In brief, formalin-fixed paraffin-embedded tumor tissue blocks were cut into 4 μm thick sections. After drying, de-paraffinized, and dehydrated in graded ethanol, tissue sections were immersed in 3% hydrogen peroxide for 10 minutes at room temperature to block endogenous tissue peroxidase activity. The antigen was retrieved by citrate buffer (pH 6.0) and high-heat microwave processing for 5 minutes, followed by washing in PBS. All slides were incubated with primary HIF-1α antibody (YT2133, Immunoway, 1:200 dilution), VEGF-A antibody (YT5108, Immunoway, 1:200 dilution), and Ki67 antibody (SP6, DAKO, ready-to-use) overnight at 4°C, followed by incubation for 30 minutes in Ultra-Sensitive SP kit (Maixin-Bio, Fuzhou, China). The slides were rinsed with phosphate-buffered saline before color development using a 3, 3’-diaminobenzidine substrate kit and were then counterstained with hematoxylin.

### 2.4. Evaluation of Immunohistochemistry

Two independent senior pathologists who were blinded to the clinicopathological data examined the immunoreactivity of HIF-1α, VEGF-A, and Ki67. A total of 10 areas were randomly selected and counted at a magnification of × 200. The staining results were scored semi quantitatively based on staining intensity and percentage of positive cells; Intensity of stained cells was graded into 0 (no staining), 1 + (weak staining: light yellow), 2 + (moderate staining: yellow-brown), and 3 + (strong staining: brown); Percentage of positive cells was scored as: 0 (<5% of cells), 1 point (6%–25% of cells), 2 points (26%–50% of cells), 3 points (51%–75% of cells), and 4 points (>75% of cells). The immunoreaction score of each marker was the multiplication of intensity and percentage of positive cells. IHC staining of Ki67 was scored as the percentage of positive tumor cells to all tumor cells, such as 5%, 10%, 20%, 30%, 40%, 50%, 60%, 70%, 80%, 90%, and 100%.

### 2.5. Statistical analysis

Statistical analysis was performed using IBM SPSS Statistics, Version 21.0 (IBM Corp., Armonk, NY). Continuous variables were presented as mean ± standard deviations and non-normally distributed variables were presented as median (P25-P75). The expression of HIF-1α, VEGF-A, and Ki67 between different groups (nonneoplastic cervical epithelial tissues vs cancerous tissues, pre-NACT vs post-NACT, responders vs non-responders) was evaluated by the Wilcoxon test. The expression of HIF-1α, VEGF-A, and Ki67, as well as clinicopathological features of cervical cancer patients between the NACT responsive and nonresponsive groups was analyzed by chi-square test (Age, histological grade, and FIGO stage), Student *t* test (Tumor size), or Wilcoxon test (expression of HIF-1α, VEGF-A, and Ki67). Factors affecting response to NACT were analyzed by Logistic regression. *P* values < .05 (2-tailed) were considered statistically significant.

## 3. Results

### 3.1. HIF-1α, VEGF-A, and Ki67 were overexpressed in LACC

**Figure 1.**
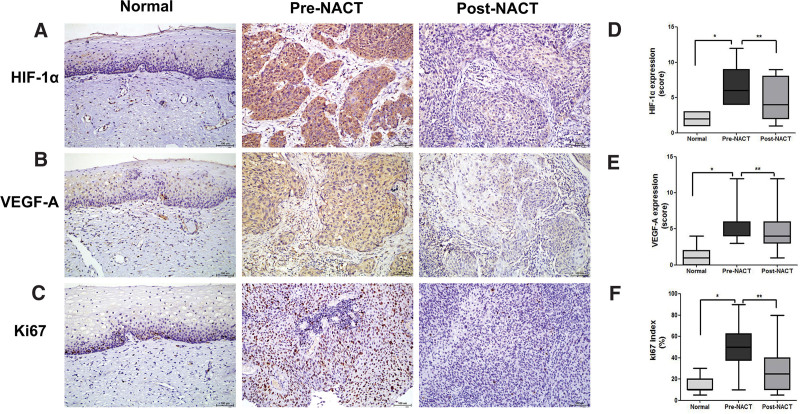
Expression of HIF-1α, VEGF-A, and Ki67 was mainly in LACC tumor cells pre- and post-NACT and basal layer cells of normal cervix squamous epithelium by IHC (SP staining, × 200). HIF-1α protein, (A) was detected primarily in the cytoplasm and partially in the nuclei; VEGF-A protein, (B) was detected primarily in the cytoplasm; and Ki67, (C) was detected primarily in the nuclei. Scores for HIF-1α, (D) and VEGF-A, (E) staining and Ki67 index, (F) in basal layer cells of normal cervical tissues vs LACC pre- and post-NACT. Asterisks (* and **) indicate *P* < .05. HIF-1α = hypoxia-inducible factor 1α, IHC = immunohistochemical, LACC = locally advanced cervical cancer, NACT = neoadjuvant chemotherapy, VEGF-A = vascular endothelial growth factor.

The expression of HIF-1α, VEGF-A, and Ki67 in 42 cases of LACC and 40 nonneoplastic cervical epithelial tissues was investigated by IHC. Cytoplasmic or nuclei expression of HIF-1α and VEGF-A and nuclei expression of Ki67 were observed mainly in the tumor cells of LACC samples. Regarding nonneoplastic tissues, there was a weak or no staining of HIF-1α, VEGF-A, and Ki67 in the basal layer of cervix squamous epithelium (Fig. 1A–C). As shown in Figure 1, HIF-1α, VEGF-A, and Ki67 proteins were overexpressed in LACC tissues compared to nonneoplastic tissues. Semi-quantification of HIF-1α, VEGF-A, and Ki67 staining showed that their immunoreactive scores were much higher in LACC compared to nonneoplastic tissues (*P* < .01, respectively) (Table 1, Fig. 1D–F).

**Table 1:** The expression of HIF-1α and VEGF-A proteins in the basal layer cells of adjacent normal and tissues tumor cells of cervical cancer pre- chemotherapy.

Group	n	HIF-1α	VEGF-A	Ki67 (%)
Normal basal cell	40	2.00 (1.00–3.00)	1.00 (0.00–2.00)	10 (10–20)
Cancer cell Pre-chemotherapy	42	6.00 (4.00–9.00)	6.00 (4.00–6.00)	50 (37.5–62.5)
Z Value		−7.888	−7.789	−7.163
*P* value		<.001	<.001	<.001

HIF-1α = hypoxia-inducible factor 1α, VEGF-A = vascular endothelial growth factor.

### 3.2. Expression of HIF-1α, VEGF-A, and Ki67 was decreased after NACT in LACC

Expression of HIF-1α, VEGF-A, and Ki67 was determined in 42 primary LACC biopsies before NACT as well as in their matched specimens after NACT. Pre-chemotherapy cervical cancer biopsies showed strong positive staining of HIF-1α, VEGF-A, and Ki67, while weak or moderate positive staining was seen in post-chemotherapy specimens (Fig. 1A–C). The expression of HIF-1α, VEGF-A, and Ki67 was significantly higher in pre-chemotherapy tissues compared to post-chemotherapy tissues (*P* < .01, respectively) (Table 2, Fig. 1D–F), suggesting that HIF-1α, VEGF-A, and Ki67 expression was decreased after NACT in LACC.

**Table 2:** The expression of HIF-1α, VEGF-A and Ki67 proteins in tumor cells of cervical cancer pre- and post- chemotherapy.

Group	n	HIF-1α	VEGF-A	Ki67
Cancer cell Pre-chemotherapy	42	6.00 (4.00–9.00)	6.00 (4.00–6.00)	50 (37.5–62.5)
Cancer cell Post-chemotherapy	42	4.00 (2.00–8.00)	4.00 (3.00–6.00)	25 (10–40)
*Z* value		−3.231	−2.799	−4.610
*P* value		.001	.005	<.001

HIF-1α = hypoxia-inducible factor 1α, VEGF-A = vascular endothelial growth factor.

### 3.3. Decreasing expression of HIF-1α, VEGF-A, and Ki67 was associated with response to NACT in LACC

**Figure 2.**
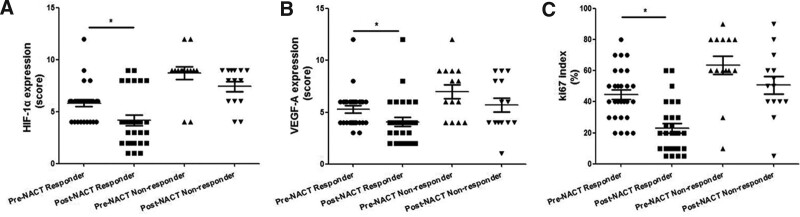
HIF-1α, VEGF-A, and Ki67 expression in pre- and post-chemotherapy LACC tissues between the response group and the nonresponse group. In the response group, HIF-1α (A), VEGF-A (B), and Ki67 (C) expression were significantly decreased in the post-chemotherapy cervical cancer tissues compared with the pre-chemotherapy cervical cancer tissues; While the same was not seen in the nonresponse group. HIF-1α = hypoxia-inducible factor 1α, LACC = locally advanced cervical cancer, VEGF-A = vascular endothelial growth factor.

To explore the efficacy of NACT, the expression of HIF-1α, VEGF-A, and Ki67 in pre-chemotherapy and post-chemotherapy cervical cancer tissues between the chemotherapy response (CR + PR) group and the nonresponse (SD + PD) group were examined by IHC. Clinical response was achieved in 28 (66.7%) of the patients, including 16 (57.1%) with CR and 12 (42.9%) with a PR; While 14 (33.33%) were non responders, including 6 (42.9%) with SD and 8 (57.1%) with PD. In the response group, HIF-1α, VEGF-A, and Ki67 expression were significantly decreased in the post-chemotherapy cervical cancer tissues compared with the pre-chemotherapy cervical cancer tissues (*P* < .05, respectively); While in the nonresponse group, HIF-1α, VEGF-A, and Ki67 expression showed no significant difference in the post-chemotherapy cervical cancer tissues compared with the pre-chemotherapy cervical cancer tissues (all *P* > .05) (Table 3, Fig. 2). This indicated that decreasing expression of HIF-1α, VEGF-A, and Ki67 expression was associated with the response to chemotherapy.

**Table 3:** The expression of HIF-1α, VEGF-A and Ki67 proteins between pre- and post- chemotherapy in response and nonresponse group.

	Responders (n = 28)		*P* value	Non responders (n = 14)		*P* value
	Pre-chemotherapy	Post-chemotherapy		Pre-chemotherapy	Post-chemotherapy	
HIF-1α expression	6.00 (4.00–6.00)	4.00 (2.00–7.00)	.009	9.00 (9.00–9.00)	8.00 (5.50–9.00)	.114
VEGF-A expression	6.00 (4.00–6.00)	4.00 (2.00–5.50)	.030	7.00 (4.00–9.00)	5.00 (4.00–8.25)	.188
Ki67 (%)	40.0 (30.0–57.5)	20.0 (10.0–30.0)	<.001	60.0 (60.0–80.0)	50.0 (40.0–62.5)	.126

HIF-1α = hypoxia-inducible factor 1α, VEGF-A = vascular endothelial growth factor.

### 3.4. Factors affect the efficacy of NACT in LACC

Factors including tumor size, age, and histological grade as well as HIF-1α, VEGF-A, and Ki67 expression, affecting the efficacy of NACT were analyzed. It was shown that histological grade (*P* = .002), HIF-1α (*P* < .001), VEGF-A (*P* = .034), and Ki67 (*P* = .009) were all risk factors, while tumor size, age, and FIGO stage were not risk factors affecting the efficacy of NACT (all *P* > .05) (Table 4). Considering the above factors, a multivariate logistic regression model was used to evaluate the factors affecting response to NACT. As shown in Table 5, results showed that histological grade [*P* = .025, HR (95% CI): 0.133 (0.023–0.777)], HIF-1α [*P* = .019, HR (95% CI): 0.599 (0.390–0.918)] and Ki67 [*P* = .036, HR (95% CI): 0.946 (0898–0.996)] are independent risk factors affecting the efficacy of NACT.

**Table 4:** The clinicopathological features and expression of HIF-1α, VEGF-A and Ki67 in cancer cells of pre-chemotherapy cervical cancer between chemotherapy response and nonresponse group.

	Total no of patients	Responders (n = 28)	nonresponders (n = 14)	Response rate (%)	*P* value
Age					
<50	22	12	10	54.5	.081
≥50	20	16	4	80.0	
Tumor size (cm)	42	3.729 ± 1.124	4.057 ± 0.788	66.67	.280
Histological grade					
G1	15	14	1	93.3	.002
G2	17	12	5	70.6	
G3	10	2	8	20.0	
FIGO					
IB2	10	10	0	100.0	.149
II	20	10	10	50.0	
III	12	8	4	75.0	
HIF-1α expression	42	6.00 (4.00–6.00)	9.00 (9.00–9.00)	66.7	<.001
VEGF-A expression	42	6.00 (4.00–6.00)	7.00 (4.00–9.00)	66.7	.034
Ki67 (%)	42	40.0 (30.0–57.5)	60.0 (60.0–80.0)	66.7	.009

FIGO = International Federation of Gynecology and Obstetrics, HIF-1α = hypoxia-inducible factor 1α, VEGF-A = vascular endothelial growth factor.

**Table 5:** Logistic regression analysis of factors affact neoadjuvant chemotherapy for cervical cancer.

	β	SE	Wald	*P* value	HR (95% CI)
Histological grade	−2.018	0.901	5.015	.025	0.133 (0.023–0.777)
HIF-1α expression	−0.513	0.218	5.522	.019	0.599 (0.390–0.918)
VEGF-A expression	−0.137	0.248	0.307	.580	0.872 (0.537–1.416)
Ki67 (%)	−0.055	0.026	4.405	.036	0.946 (0898–0.996)

HIF-1α = hypoxia-inducible factor 1α, VEGF-A = vascular endothelial growth factor.

## 4. Discussion

Cervical cancer is a clinical and pathological heterogeneous malignancy with different treatment strategies, which result in a variety of outcomes. Surgery and radiochemotherapy are accepted as the standard treatment for cervical cancer.^[3]^ In China, LACC accounts for > 80% of cervical carcinoma, and most LACC are highly sensitive to platinum and paclitaxel.^[22]^ NACT was suggested for the control of the disease before surgery and has been widely used for LACC for decades.^[3]^

In a large majority of LACC patients treated with NACT, the treatment could shrink the tumor size and suppress micrometastases, thus improving prognosis and reducing the risk of recurrence.^[6,7]^ However, for some patients who have no response to NACT, the treatment strategy must be changed to radiation therapy since a hysterectomy cannot be performed, thus might result in a long period of treatment delay as well as a worse prognosis.^[8,23]^ Therefore, screening out factors that could evaluate the response to NACT will be helpful in LACC patients who choose NACT. On the one hand, sorting out those patients who will be responsive to NACT could then provide them with prompt and personalized treatment; On the other hand, identifying non responders could refrain these patients from receiving unnecessary treatment and turn to more effective treatments as soon as the disease is diagnosed.

Hypoxia, which is caused by the rapid and uncontrolled proliferation of tumor cells, contributes to the heterogeneity of tumors and could promote a more aggressive and metastatic phenotype in nearly all solid tumors.^[24]^ HIF-1α is a predominant regulator of intratumoral hypoxia and controls a series of hypoxia-mediated pathological processes in tumors, including carcinogenesis, angiogenesis, and therapy resistance.^[16]^ Previous research has indicated that HIF-1α is up-regulated in cervical cancer and that high levels of HIF-1α expression are associated with a poorer prognosis.^[25]^ In addition, the expression of HIF-1α might predict the efficacy of NACT and was considered to be an independent prognostic factor for cervical cancer.^[15]^ In line with the previous studies, our present study demonstrated that HIF-1α expression was overexpressed in LACC tissues compared to nonneoplastic tissues as well, indicating that HIF-1α played a role in tumorigenesis of LACC. In addition, the expression of HIF-1α was significantly higher in pre-chemotherapy biopsy tissues compared to post-chemotherapy specimens, suggesting that HIF-1α expression was decreased after NACT in LACC.

VEGF is one of the most important proangiogenic factors that contribute to tumor progression in numerous cancers. Chen et al^[26]^ reported that reduced expression of VEGF in cervical cancer could inhibit migration, invasion, and improve the prognosis of patients with cervical cancer. What is more, a previous study has demonstrated that the expression of VEGF and HIF-1α has great clinical significance for the prediction of preoperative radiochemotherapy response and prognosis in patients with LACC.^[19]^ Consistent with the finding of the above study, our present study found VEGF-A expression was significantly decreased in post-chemotherapy specimens compared with pre-chemotherapy biopsy tissues, suggesting that VEGF-A was decreased after NACT. Ki67 is a nuclear antigen gene that importantly reflects the proliferative activity and malignant transformation of tumors, and was significantly decreased in post-chemotherapy specimens compared with pre-chemotherapy biopsy tissues.

In addition, HIF-1α, VEGF-A, and Ki67 expression in the response group were significantly decreased in the post-chemotherapy cervical cancer tissues compared with the pre-chemotherapy cervical cancer tissues; While in the nonresponse group, HIF-1α, VEGF-A, and Ki67 expression showed no significant difference after NACT. This indicated that decreasing expression of HIF-1α, VEGF-A, and Ki67 expression was associated with a good response to chemotherapy. We further analyzed the factors that affect the efficacy of NACT and found that lower expression of HIF-1α, VEGF-A, and Ki67 could be factors affecting the efficacy of NACT. Thus, the current study suggests that HIF-1α, VEGF-A, and Ki67 may be implicated in evaluating the efficacy of NACT in LACC and measuring HIF-1α, VEGF-A, and Ki67 proteins in biopsy samples before chemotherapy may be helpful in the management of LACC.

Although the results of the current study indicate that HIF-1α, VEGF-A, and Ki67 are of potential value in evaluating the efficacy of NACT in LACC, the sample size of the study is too small and would limit the generalizability. In addition, due to the limited number of patients, the follow-up duration which reflects the prognosis is missing in the present study. Future studies with larger sample sizes are required to confirm our findings, especially studies that include prolonged follow-up to allow the analysis of diagnosis.

## 5. Conclusion

Our present study demonstrated that HIF-1α, VEGF-A, and Ki67 were frequently overexpressed in LACC, suggesting a possible role of HIF-1α, VEGF-A, and Ki67 in tumorigenesis of LACC. Furthermore, the expression of HIF-1α, VEGF-A, and Ki67 was significantly decreased after NACT, and decreasing expression of these biomarkers was related to good response to NACT. The present study indicated that the detection of HIF-1α, VEGF-A, and Ki67 in biopsy samples before chemotherapy may contribute to more effective management of LACC. However, multi-center studies with larger sample sizes and multiple approaches should be carried out in future research.

## Author contributions

**Conceptualization:** Ke Zhao, Min Hu, Tingkuan Zhao.

**Data curation:** Min Hu, Jing Liu.

**Methodology:** Ke Zhao, Runfeng Yang, Pingfan Zeng.

**Software:** Runfeng Yang.

**Writing – original draft:** Ke Zhao, Min Hu.

**Writing – review & editing:** Runfeng Yang, Jing Liu, Tingkuan Zhao.
